# Mediating Role of Self-Efficacy in the Association Between Fatigue and Depressive Symptoms in Females with Rheumatoid Arthritis

**DOI:** 10.3390/medicina61061013

**Published:** 2025-05-29

**Authors:** I-Yu Hsiao, Hanoch Livneh, Chieh-Tsung Yen, Ming-Chi Lu, Wei-Jen Chen, Tzung-Yi Tsai

**Affiliations:** 1 Department of Nursing, Dalin Tzu Chi Hospital, Buddhist Tzu Chi Medical Foundation, Chiayi 62247, Taiwan; 2 Rehabilitation Counseling Program, Portland State University, Portland, OR 97207-0751, USA; livnehh@pdx.edu; 3 Department of Neurology, Dalin Tzu Chi Hospital, Buddhist Tzu Chi Medical Foundation, Chiayi 62247, Taiwan; 4 Division of Allergy, Immunology and Rheumatology, Dalin Tzu Chi Hospital, Buddhist Tzu Chi Medical Foundation, Chiayi 62247, Taiwan; 5 School of Medicine, Tzu Chi University, Hualien 97004, Taiwan; 6 Department of Chinese Medicine, Dalin Tzuchi Hospital, Buddhist Tzu Chi Medical Foundation, Chiayi 62247, Taiwan; 7 Graduate Institute of Sports Science, National Taiwan Sport University, Taoyuan 333325, Taiwan; 8 School of Post-Baccalaureate Chinese Medicine, Tzu Chi University, Hualien 97004, Taiwan; 9 Center of Sports Medicine, Dalin Tzuchi Hospital, Buddhist Tzu Chi Medical Foundation, Chiayi 62247, Taiwan; 10 Department of Environmental and Occupational Health, College of Medicine, National Cheng Kung University, Tainan 70428, Taiwan; 11 Department of Medical Research, Dalin Tzu Chi Hospital, Buddhist Tzu Chi Medical Foundation, Chiayi 62247, Taiwan

**Keywords:** fatigue, self-efficacy, depressive symptoms, rheumatoid arthritis, mediation

## Abstract

*Background and Objectives*: Extant research on the relationship between fatigue and depression in people with rheumatic diseases portrays a divergent picture. While caring for persons with this medical condition, one issue that represents individual confidence in carrying out specific tasks, namely self-efficacy level, has attracted significant attention. Yet, the information regarding whether self-efficacy may pose a clue linking these two major symptoms is still unknown. The aim of this study, therefore, is to examine whether self-efficacy mediates the association between fatigue and depressive symptoms among persons with rheumatoid arthritis (RA). *Materials and Method*: A cross-sectional study of 224 females with RA from a hospital in Taiwan was conducted between January and October 2023. We then distributed anonymous self-reported questionnaires instructing participants to provide information on their demographic characteristics, levels of fatigue, self-efficacy, and depressive symptoms. The bootstrap via PROCESS macro in SPSS was executed to analyze if self-efficacy would mediate the effect of fatigue on emergence of depressive symptoms. *Results*: For those participants captured at baseline, a negative association was noted between fatigue and self-efficacy, as well as between self-efficacy and depressive symptoms. Results of the mediation analysis revealed a remarkable indirect effect of fatigue on depressive symptoms through self-efficacy, with a regression coefficient of 0.21 (95% confidence intervals: 0.06–0.37). *Conclusions*: This work extends current understanding of the roles that fatigue and self-efficacy play in predicting depression among people with RA and further clarified the potential mediating role of self-efficacy in buffering against depressive symptomatology. Interventions that extend from the management of fatigue and further incorporate the improvement of self-efficacy sense into the stereotypical therapy should greatly mitigate the distressing symptoms for patients with RA.

## 1. Introduction

A growing body of evidence has been aggregated to highlight that chronic inflammation may play a decisive role in many pathophysiological conditions [1]. Take rheumatoid arthritis, often abbreviated as RA, as an example. It is a progressive form of joint destruction that affects millions of people worldwide, particularly women [2]. Despite the imprecise etiology of this disease, the dysregulated immune response has been assumed to be a pivotal contributor to RA attack [3,4]. In a dysfunctional immune system, several serum pro-inflammatory cytokines are released from T helper (Th) cells in RA patients [5]. Through humoral and neural routes, these inflammatory precursors may incur extra-articular manifestations, especially depression. In a recent meta-analysis of 72 studies that included a total of 13,189 RA participants, depression was indicated to be highly prevalent among them, with a reported prevalence ranging from 15 to 40% [6]. An earlier study also reported that RA patients were approximately twice as likely to experience depression compared with the general population [7]. Notably, the presence of co-occurring depression would nearly double the likelihood of death for them [8]. Other consequences of comorbid depression include greater healthcare consumption, diminished social abilities, and increased risk of vascular-related events [9,10]. Accordingly, a better understanding of the precursors of depression should speed up the implementation of effective strategies against depression attacks in at-risk populations.

Recently, the updated recommendation from the European League Against Rheumatism (EULAR) has put out a call that one critical perspective should be urgently monitored while managing RA persons, which is fatigue management [11]. Fatigue refers to an overwhelming, intrusive, and draining exhaustion of physical and mental energy, and is different than more typical tiredness in which long periods of rest or sleep are typically beneficial [12]. Despite therapies approved for RA, approximately 50% of affected patients experienced fatigue [13]. As such, current research has focused on the interplay between fatigue and depression among chronic rheumatic diseases. Yet, the relevant empirical findings are still mixed and inconclusive. One recent cross-sectional study of 474 RA patients showed that depressive symptoms strongly correlated with fatigue level as assessed by visual analog scale (VAS). The authors concluded that the interrelationship between these two conditions revolved around the roles of inflammatory precursor substances, such as interleukin (IL)-1β, IL-6, and tumor necrosis factor-α (TNF-α), all of which highly correlated with the pathogenesis of inflammatory disorders [14]. In that study, for each additional unit increase in fatigue, depression risk increased by 26% [14]. Other studies in systemic lupus erythematosus patients also revealed a positive relationship between fatigue and risk of depression [15]. However, one review article disclosed that depression cannot fully depend upon its synchronous fatigue sensation among those with rheumatic disorders, as some not-depressed but fatigued patients indeed occurred [16]. In a study investigating the association between depression and cancer-related fatigue, the authors failed to observe the directionality of fatigue to depressive symptoms [17]. Taken together, these findings imply that some hidden factors may mask the clinical process linking fatigue to risk of depressive symptoms. Such findings further uncovered that any attempts to institute intervention modalities to manage fatigue and subsequent risk of depressive symptoms may be premature unless better understanding of their casual relationships can be purified.

When caring for persons afflicted with RA, the concept of self-efficacy has drawn much attention and has been viewed as a priority, as shown in the recent EULAR experts’ recommendations [18]. Several investigations revealed a negative bond between self-efficacy and depression [19,20], and also suggested that the relief of fatigue may strengthen the individual’s self-efficacy [21]. Despite this, few studies were conducted to address the link between fatigue and depressive symptoms among RA groups, focusing on the mediating role of self-efficacy. To address this lacuna, we embarked on an exploration of the role of self-efficacy in mediating the relationship between fatigue and depressive symptoms among RA females, thus seeking to bridge the research gap between fatigue and depression and provide empirically-derived suggestions on how to manage these distressing symptoms for persons living with RA.

## 2. Materials and Methods

### 2.1. Study Design and Participants

Using females with RA from a single teaching hospital in Taiwan as the study population, this cross-sectional study was carried out between January and October of 2023. The inclusion criteria for participants included the following: (a) diagnosed with RA by a rheumatologist and adhering to criteria per the American College of Rheumatology and EULAR 2010 [22]; (b) visited the outpatient clinic of rheumatology and immunology; (c) being of the minimum age of 20 years old at the time of diagnosis; and (d) capable of understanding Mandarin. Subjects who met these criteria were invited to participate in this study. Those with psychiatric diagnoses or an inability to communicate were removed. The sample size was estimated post hoc using G*Power Version 3.1. Based on the sample size of females with RA recruited by end of study, the statistical power was estimated to be 0.94, which indicated that the number of enrollees fulfilled the desired statistical rule.

### 2.2. Measurements

We capitalize on structured questionnaires to measure some indicators that centered on self-efficacy, fatigue, and depressive symptoms. Self-efficacy was estimated using the Arthritis Self-Efficacy Scale (ASES) that was developed by Lorig and colleagues in 1980 [23]. This scale has been widely used for patients with rheumatic diseases and comprised 20 items that measure three domains (pain, function, and other symptoms). Each of which is scored from 10 to 100, with higher scores representing a greater degree of self-efficacy [23]. This scale has been translated into the Chinese version of ASES (C-ASES) by Tsai and colleagues to suit the Chinese cultural setting [24]. Cronbach’s alpha for the total C-ASES in the present study was 0.92.

The level of depressive symptoms was determined using the Taiwanese Depression Questionnaire (TDQ) as it exactly reflects Asian culture and is written in the Taiwanese language [25]. The TDQ consisted of 18 items and scored on a four-point Likert-type scale ranging from 0 (absence of symptoms) to 3 (presence of symptoms almost every day) with a recall period over the past week. The total score, therefore, ranged from a minimum score of 0 to a maximum score of 54. By applying the structured clinical interview as the gold standard, TDQ possessed an acceptable concurrent validity, with the received operating characteristics curve of 0.92 [26]. Other studies that have employed the TDQ showed good internal consistency for persons living with chronic diseases, with Cronbach’s alpha coefficient ranging from 0.89 to 0.92 [27,28]. Cronbach’s alpha for the current study participants was 0.95. As for the level of fatigue, it was measured via a self-reported visual analog scale (VAS) that has been extensively utilized and validated across the rheumatic disease spectrum [29]. VAS is often employed to distinguish the intensity and frequency of subjective experiences on an eleven-point numerical scale, ranging from 0 to 10, where higher scores implicate a more severe degree of experienced fatigue [30]. Presently, it has been shown to be a psychometrically sound measure for patients with musculoskeletal pain [31].

The second part of the questionnaire contained information on demographic and disease characteristics, all of which were developed from previous studies and clinical experience [32]. The demographic data comprised participants’ age, marital status, education level, monthly income, living status, and lifestyle factors that included smoking and exercise habits. Information regarding smoking was dichotomously divided into smokers (those who reported smoking at least one package per week) or non-smokers. Study participants were categorized as regular exercisers if they exercised for at least 20 min, three times per week. Disease characteristics included comorbid conditions such as diabetes mellitus, hypertension, heart disease, stroke, or cancer. Several features closely linked with this disease were also included, such as disease activity score in 28 joints (DAS28), body mass index (BMI), duration of RA, C-reactive protein (CRP), and prescriptions of biological disease-modifying anti-rheumatic drugs (DMARDs). Participants were also asked if they had taken biological DMARDs for more than 3 months due to RA (e.g., etanercept, adalimumab, infliximab, or rituximab). A review of medical records was used to assess the aforesaid disease-related characteristics.

### 2.3. Procedure of Data Gathering

Approval for this study was granted by the institutional review board at the Buddhist Dalin Tzu Chi Hospital on 31 January 2023 (No. B11201014). At the beginning of the study, the researchers explained the purpose of the study and the relevant procedure. Informed consent was obtained after the participants fully understood and agreed to take part in the study. During the survey administration period, the researchers were available to answer any inquiries. Those who were unable to complete the questionnaires in the allotted time, at the hospital setting, were allowed to do so at home (returned via mail within 7 days in pre-stamped anonymous envelopes). Throughout the study, subjects had the option to withdraw at any time if needed.

### 2.4. Data Analysis

All statistical analyses in this study were conducted using SPSS 22.0 (Chicago, IL, USA) and the SPSS PROCESS macro (Version 4.2). First, participants’ personal characteristics and measures of fatigue, self-efficacy, and depressive symptoms are reported with descriptive statistics (relevant data were expressed as frequency [percentage] for ordinal and categorical data, or as means and standard deviations [SD] for continuous data). Pearson’s correlations were applied to analyze the correlations between fatigue, self-efficacy, and depressive symptoms. The mediating effect of self-efficacy between fatigue and depression was examined using the PROCESS macro Version 4.2 for SPSS [33]. The outputs of the PROCESS macro were organized as follows: (1) self-reported fatigue could significantly predict depressive symptoms in a regression equation (estimation and test path c); (2) self-reported fatigue significantly associated with self-efficacy in a regression equation (estimation and test path a); (3) self-efficacy attitude notably predicted depressive symptoms in the regression equation (estimation and test path b); and (4) indirect effect of self-efficacy on fatigue and depression (X through M to Y, path a*b). One null hypothesis was then set in which it represented the mediator of interest exited once the indirect effect of a*b did not equal zero. The coefficient c′ indicates the direct effect of fatigue on depressive symptoms after controlling self-efficacy influence on depressive symptoms. All of the aforesaid indicators, including estimates of a, b, c, and c′, were used to establish the schematic figure. At the same time, intermediation hypotheses were tested with the bias-corrected bootstrap method with 5000 samples to calculate confidence intervals (95% CIs). An indirect effect was considered to be significant when the 95% CIs range does not span zero [33].

## 3. Results

### 3.1. Differences of Depressive Symptoms at Baseline by Demographic and Disease Features

The study cohort (*n* = 224 subjects) had a mean age of 51.8 ± 13.6 years. Most participants were married (79.5%), cohabitating (85.7%), and reported achieving a lower educational level (<9th grade) (54.0%). The majority of enrollees were non-smokers (96.9%), reported higher monthly income (71.9%) (≥30001 NTD), and followed a regular exercise routine (50.9%). The mean duration of RA was 11.8 years, and the mean BMI and DAS28 were 25.78 and 3.58, respectively. Over 60% of the subjects suffered from comorbid conditions (61.2%) and received biological agents (61.6%), as shown in Table 1.

### 3.2. Correlations Between Fatigue, Self-Efficacy, and Depressive Symptom

Relationships between fatigue, self-efficacy, and depressive symptoms were estimated by Pearson’s correlation coefficients (Table 2). Greater fatigue was negatively associated with self-efficacy level and positively related to depressive symptoms (r = −0.50, *p* < 0.001; r = 0.35, *p* < 0.001). In addition, self-efficacy level was negatively associated with depressive symptoms (r = −0.36, *p* < 0.001).

### 3.3. Analysis of Mediation Effect

**Figure 1:**
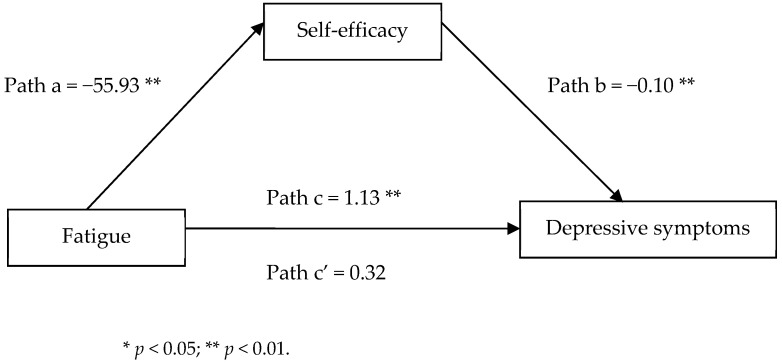
Mediation analysis of fatigue to the sequent depressive symptoms through self-efficacy.

Prior to the testing for mediating effect, individual DAS28 and comorbidity conditions shown in the univariate analysis were considered as covariates. The total effect of fatigue on depression was found to be significant (path c: *B* = 1.13, *p* = 0.003) after adjusting for the effect of covariates. The direct effects of fatigue on self-efficacy (path a: *B* = −55.93, *p* < 0.001) and of self-efficacy on depression (path b: *B* = −0.10, *p* = 0.001) were both significant after adjusting for covariates. The direct effect of fatigue on depressive symptoms did not reach statistical significance after controlling for mediators and covariates (path c′: *B* = 0.32, *p* = 0.42), which suggests that the mediation effect is indeed present. Furthermore, the indirect impact of self-efficacy reached statistical significance since the 95% CI range did not include zero (path a*b = 0.54, 95% Cis = 0.22–0.81), thus suggesting that self-efficacy completely mediated the relation between fatigue and depressive symptoms among the enrollees (Table 3 and Figure 1).

## 4. Discussion

Despite acknowledging the higher prevalence of depression among RA individuals, the possible contributing factors to depression still remain under-researched, especially the role of fatigue. The association between fatigue and subsequent depressive symptoms using hospital/community-based populations with rheumatic disorders has been examined in some studies [14,15,16], but the findings portrayed an inconclusive picture. To top it all off, earlier studies appear to ignore the occurrence of mediators that might harbor the crosstalk between fatigue and depression.

The current study is the first to explore the mediating effect of self-efficacy in linking fatigue and depressive symptoms among persons living with RA. We noted that fatigue was significantly associated with lower self-efficacy levels, and self-efficacy was positively tied to lower depressive symptoms, both of which were consistent with prior reports [19,20,21]. Actually, the intermediate role of self-efficacy in depressive symptoms has been addressed in the diverse study groups [34,35]. Herein, we observed that self-efficacy appeared to be imperative in the cause-effect linkage between fatigue and depression in RA females. Consequently, among individuals with RA with a strong sense of self-efficacy, the intrinsic fatigue would not pertain to subsequent risk of depressive symptoms. In contrast, even though they had a mild fatigue level, they still encountered a greater chance of depression upon experiencing a weak sense of self-efficacy. Our findings are consistent with previous reports showing that lower self-efficacy is associated with more severe psychological distress [18,19,34]. Not only bridging the chasm pertaining to the association of fatigue with the emergence of depressive symptoms, our study unfolded that the management of depressive symptoms in RA persons goes beyond simply the relief of fatigue level; instead, the improvement of self-efficacy needs to be stressed in the early stages of the care.

To successfully live with any chronic disease, the affected person must continuously learn about the illnesses and practice relevant self-management behaviors to engage in daily care affairs. More recently, several nontraditional methods of improving self-efficacy have been reported, such as digital technologies and self-care management [36]. Digital technologies refer to a set of digital artifacts supported by digital tools and systems, like social media for communication or joining e-learning platforms [37]. A randomized controlled trial was carried out with 180 patients with RA, and higher self-efficacy was reported for those who completed an e-learning education course as compared with those who received face-to-face traditional education [38]. However, considering that elderly people may have limited access to online health education resources, as well as limited internet use proficiency [39], it may be suggested to tailor the individual strategies to the local infrastructure, thus maximizing the motivation to strictly adhere to the therapeutic paradigms.

As for the benefits of the self-care management program, our prior work had revealed that an embedded nurse-led case management program may yield benefits for RA patients, and in particular for self-efficacy [28]. The benefits of that program persisted for six months after completion of the self-care management program [28]. Implementation of such a program by nursing care staff through one-on-one consultation and regular follow-up may ensure that patients take an active role in disease management. A highly interactive manner and the use of colored images utilized in nurse-led case management may be beneficial in engaging patients and assisting them in making appropriate plans for disease management, thereby strengthening self-efficacy to combat distressing symptoms generated by the medical conditions. Moreover, a freeware web-based platform may be considered to allow patients to transmit data and receive timely feedback to identify any potential issues [28], thus gradually bolstering intrinsic self-efficacy and treatment compliance [40].

While our study is the first to investigate the mediating role of self-efficacy in linking fatigue and depressive symptoms among people with RA, there are important limitations to consider. First, the reported findings are derived from a sample of patients at a single hospital. Though it is not uncommon for studies to draw conclusions from small and arbitrary sample sizes, a potentially non-representative sample might have limited the ability to generalize the study findings. Future investigations of this concern, via the use of various recruitment methods in ethnically diverse populations, are therefore warranted. Second, since we implemented a cross-sectional design, there is limited information about any longitudinal and fluctuating trajectories of depressive symptoms over time. Longitudinal research would allow researchers to shed light on any causal relationships among the independent, dependent, and mediating variables. Third, the self-administrated measures may be hampered by recall bias as well as social desirability, thereby possibly distorting the validity of the research outcomes. Nonetheless, recent evidence suggested the existence of a strong empirical agreement between data yielded by self-reported scales and objective findings from medical records among patients diagnosed with RA [41]. Furthermore, this method is inexpensive and relatively simple to administer for large population-based samples. Finally, this study mainly focused on females and did not control the several confounders, such as dietary intake, social network used, and laboratory-derived data. Future studies based on a larger population with rheumatic diseases are needed to validate the veracity of the mediating role of self-efficacy in this context after taking into account the possible roles played by the aforementioned confounding factors.

## 5. Conclusions

The aim of this study is to examine how self-efficacy mediates the relationship between fatigue and depressive symptoms. Our findings indicate that the influence of fatigue on depressive symptoms would be greatly mediated through the role of self-efficacy in this sample of RA subjects. As a result, the reinforcement of individual self-efficacy may bring positive therapeutic impacts while caring for persons afflicted with RA. As far as clinical practice goes, it is up to healthcare providers to take the initiative in assessing patients’ self-efficacy throughout the process of disease management. Beyond that, the practitioner can encourage RA patients to take control of their individual disability by equipping them with self-management strategies and assistive products if they need them. Also of importance, the enhancement of patients’ self-efficacy sense needs a fresh perspective at the moment of truth, and blending the digital affordances of the information sharing model into the novel multidisciplinary care is highly recommended.

## Figures and Tables

**Table 1:** Differences in depressive symptom level by demographic and disease features captured at baseline (*n* = 224).

Variables	Mean ± SD	*n* (%)	TDQ Score	
			Mean ± SD	t/r
Demographic data				
Age (years)	51.84 ± 13.65			r = 0.011
Married status				
Single		46(20.5)	14.47 ± 10.32	t = −1.53
Married		178(79.5)	17.36 ± 11.64	
Educational level				
Low (<9th grade)		121(54.0)	14.88 ± 10.46	t = −1.17
High (>9th grade)		103(46.0)	16.62 ± 11.51	
Monthly income				
≤30000 NTD		63(28.1)	15.60 ± 11.32	t = −0.17
≥30001 NTD		161(71.9)	15.88 ± 10.45	
Living status				
Living alone		32(14.3)	19.12 ± 11.59	t = −1.91
Cohabitating		192(85.7)	15.11 ± 10.89	
Cigarette smoking				
Yes		7(3.1)	16.57 ± 15.02	t = −0.30
No		217(96.9)	15.33 ± 10.77	
Regular exercise				
Yes		114(50.9)	15.44 ± 12.14	t = 0.99
No		110(49.1)	13.99 ± 9.56	
**Disease characteristics**				
Disease duration (years)	11.8 ± 5.6			r = 0.27
Comorbidity				
Yes	137(61.2)		17.76 ± 11.12	t = −3.66 **
No	87(38.8)		14.93 ± 10.76	
Biological agents				
Yes	138(61.6)		16.15 ± 11.25	t = −0.80
No	86(38.4)		14.93 ± 10.76	
BMI	25.78 ± 4.67			r = 0.03
DAS28	3.58 ± 1.35			r = 0.33 **

SD: Standard Deviation; BMI: Body Mass Index; NTD: New Taiwan Dollar; DAS 28: Disease Activity Score 28. * *p* < 0.05; ** *p* < 0.01.

**Table 2:** Relationships between fatigue, self-efficacy, and depressive symptoms (*n* = 224).

Variables	Mean	SD	Fatigue	Self-Efficacy	Depressive Symptoms
Fatigue	4.67	3.38	1	-	-
Self-efficacy	1290.53	328.6	−0.50 **	1	-
Depressive symptoms	16.79	10.06	0.35 **	−0.36 **	1

* *p* < 0.05; ** *p* < 0.01.

**Table 3:** PROCESS model summaries for mediation analysis.

Variables	Path c		Path c′ and b		Path a		Path a*b	
	β Coeff	SE	β Coeff	SE	β Coeff	SE	β Coeff	95% CIs
Fatigue	1.13	0.19 **	0.32	0.24	−55.93	8.52 **		
Self-efficacy	--	--	−0.10	0.03 **	--	--	0.21	0.06–0.37
DAS28	2.19	0.47 *	2.08	0.58	−29.64	16.51 *		
Comorbidity	1.01	0.31	1.32	0.37	−18.89	19.11		

* *p* < 0.05; ** *p* < 0.01. SE: standard error; CIs: confidence intervals.

## Data Availability

Data are available upon reasonable request.
